# Urinoma as a complication of cervical cancer

**DOI:** 10.1590/2175-8239-JBN-2024-0113en

**Published:** 2024-09-16

**Authors:** Janio de Paula Santos, Rangel de Sousa Costa, Diogo Goulart Corrêa

**Affiliations:** 1 Universidade Federal Fluminense, Hospital Universitário Antônio Pedro, Departamento de Radiologia, Niterói, RJ, Brazil.

**Figure 1:**
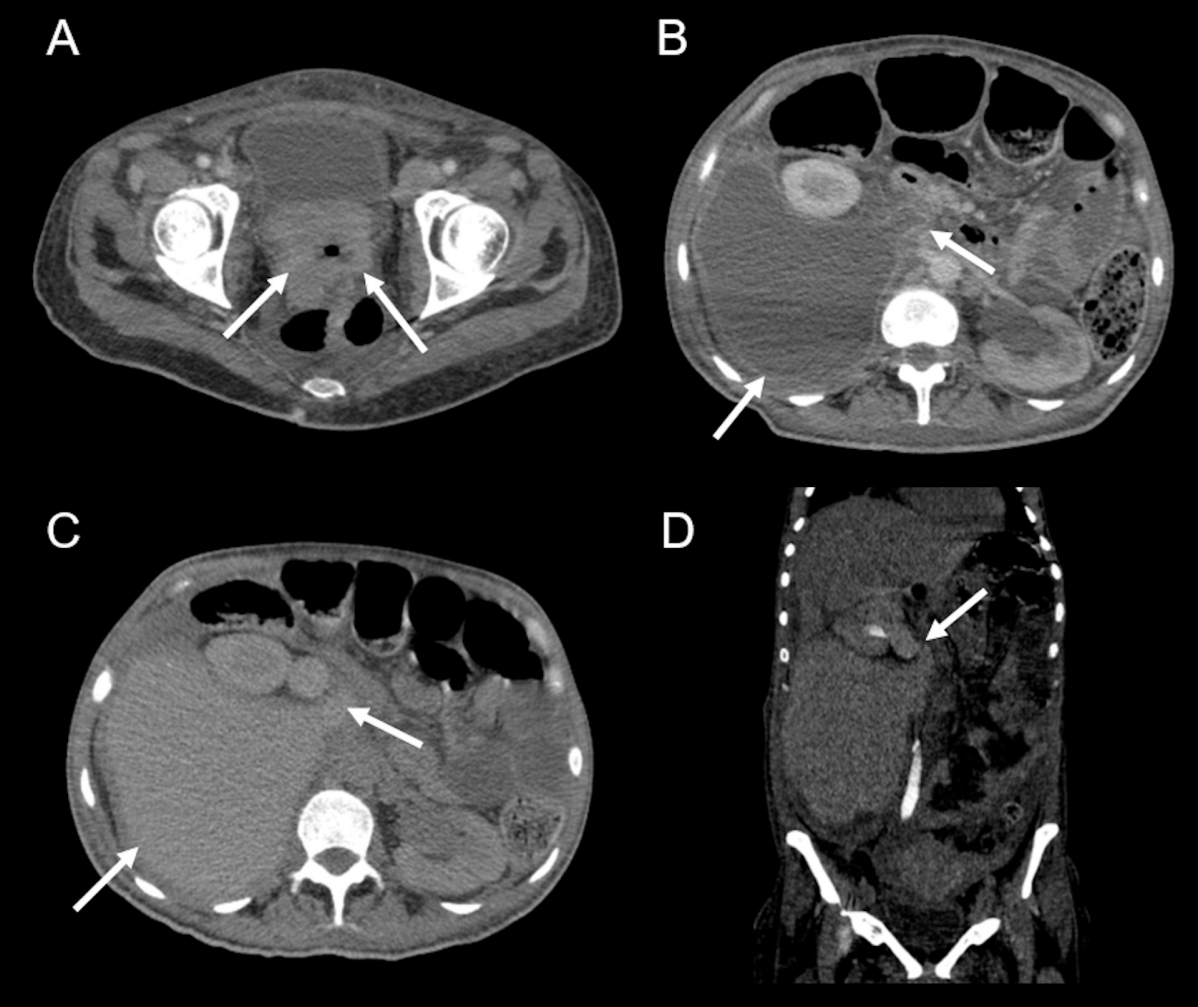
Urinoma due to rupture of the right ureter, secondary to obstruction due to cervical cancer. Computed tomography with iodinated contrast media showed, in the portal phase, thickening of the uterine cervix wall with the contrast-enhancement, invading the urinary bladder trigone (arrows in A), associated with bilateral hydronephrosis and a retroperitoneal fluid collection adjacent to the right ureter (arrows in B). In the excretory phase of the computed tomography, there was extravasation of the contrast media from the right ureter into the fluid collection (arrows in C). Reconstruction in the coronal plane of the exam better demonstrated the communication between the right ureter and the fluid collection, characterizing the urinoma (arrow in D).

A 38-year-old woman with cervical cancer presents with abdominal pain and a palpable mass in the right flank. Abdominal computed tomography demonstrated a retroperitoneal fluid accumulation communicating with the right ureter, with extravasation of contrast media from the ureter into the accumulated fluid, suggestive of urinoma secondary to ureter rupture (Figure 1). A urinoma results from leakage of urine from the urinary system due to trauma, iatrogenesis, infection, or rupture from pressure of the urogenital system^1^, whose formation requires the maintenance of renal function^2^. Early diagnosis through imaging exams is important to avoid complications such as hydronephrosis or abscess^3^.
