# Supplementary material for: Urinoma as a complication of cervical cancer
Source: J Bras Nefrol. 2024 Sep 16;46(4):e20240113. doi: 10.1590/2175-8239-JBN-2024-0113en (PMC11542636; doi:10.1590/2175-8239-JBN-2024-0113en)
Supplement: Translation

# Translation: Urinoma como complicação de câncer do colo uterino

**DOI:** 10.1590/2175-8239-JBN-2024-0113pt

**Authors:** Janio de Paula Santos, Rangel de Sousa Costa, Diogo Goulart Corrêa

**Affiliations:** 1 Universidade Federal Fluminense, Hospital Universitário Antônio Pedro, Departamento de Radiologia, Niterói, RJ, Brasil.

Mulher, 38 anos, com câncer de colo uterino apresenta dor abdominal e massa palpável no flanco direito. Tomografia computadorizada de abdome demonstrou coleção retroperitoneal, em comunicação com ureter direito, com extravasamento do meio de contraste do ureter para a coleção, sugestivo de urinoma, secundário a rompimento do ureter (Figura 1). Urinoma é o resultado do extravasamento de urina do sistema urinário, relacionado a trauma, iatrogenia, infecções ou rotura pela pressão da obstrução do sistema geniturinário^1^, cuja formação exige manutenção da função renal^2^. O diagnóstico precoce, por exames de imagem, é importante para evitar complicações, como hidronefrose ou formação de abscesso^3^.

**Figura 1:** Urinoma por rotura do ureter direito, secundário à obstrução por câncer de colo uterino. Tomografia computadorizada com contraste, na fase portal, demonstrou espessamento da parede do colo uterino, com realce pelo meio de contraste, invadindo a região do trígono vesical (setas em A), associado à hidronefrose bilateral e a uma coleção retroperitonial, com densidade líquida, adjacente ao ureter direito (setas em B). Na fase excretora da tomografia computadorizada, houve extravasamento do meio de contraste do interior do ureter direito para o interior da coleção (setas em C). A reconstrução no plano coronal do exame demonstrou mais adequadamente a comunicação do ureter direito com a coleção, caracterizando o urinoma (seta em D).
